# Changing media depictions of remote consulting in COVID-19: analysis of UK newspapers

**DOI:** 10.3399/BJGP.2020.0967

**Published:** 2020-12-15

**Authors:** Gilly Mroz, Chrysanthi Papoutsi, Alex Rushforth, Trisha Greenhalgh

**Affiliations:** Nuffield Department of Primary Care Health Sciences, University of Oxford, Oxford.; Nuffield Department of Primary Care Health Sciences, University of Oxford, Oxford.; Nuffield Department of Primary Care Health Sciences, University of Oxford, Oxford.; Nuffield Department of Primary Care Health Sciences, University of Oxford, Oxford.

**Keywords:** general practice, remote consultation, media analysis, COVID-19

## Abstract

**Background:**

Remote consulting was introduced quickly into UK general practice in March 2020 as an emergency response to COVID-19. In July 2020, ‘remote-first’ became long-term government policy.

**Aim:**

To explore how this change was portrayed in national newspapers and how depictions changed over time.

**Design and setting:**

Thematic analysis of newspaper articles referring to remote GP consultations from two time periods: 2 March–31 May 2020 (period 1) and 30 July–12 August 2020 (period 2).

**Method:**

Articles were identified through, and extracted from, LexisNexis Academic UK. A coding system of themes and narrative devices was developed and applied to the data. The analysis was developed iteratively, amending the coding structure as new data were added.

**Results:**

Remote consulting was widely covered in newspapers. Articles in period 1 depicted it positively, equating digital change with progress and linking novel technological solutions with improved efficiency and safety (for example, infection control) in a service that was overdue for modernisation. Articles in period 2 questioned the persistence of a remote-first service now that the pandemic was waning, emphasising, for example, missed diagnoses, challenges to the therapeutic relationship, and digital inequalities.

**Conclusion:**

As the first wave of the pandemic came and went, media depictions of remote consulting evolved from an ‘efficiency and safety’ narrative to a ‘risks, inequalities, and lack of choice’ narrative. To restore public trust in general practice, public communication should emphasise the wide menu of consulting options now available to patients and measures being taken to assure safety and avoid inequity.

## INTRODUCTION

The shift from in-person to remote-by-default consulting in UK general practice, introduced in March 2020 as part of the infection control measures for the first wave of the COVID-19 pandemic,^1^ was arguably the fastest and most extensive scale-up of a major service innovation in the NHS since 1948. Clinical assessment of patients shifted almost overnight to online or telephone triage, followed by telephone or video call-back and a face-to-face consultation only in rare circumstances;^2^ one large practice, for example, observed a 92.5% decrease in face-to-face consultations between the beginning and end of March 2020, and a corresponding increase in telephone and e-consultations.^3^

Many assumed that these arrangements would be temporary. Measures were gradually relaxed as the incidence of acute COVID-19 fell, resulting in 50% of consultations in England being face-to-face by July 2020.^4^ But, in late July 2020, the Secretary of State for Health and Social Care, Matt Hancock, announced that ‘remote-by-default’ consultations would remain policy even after the pandemic had receded.^5^

While the academic and clinical literature has extensively covered remote consultations (see Discussion), to the authors’ knowledge, no previous studies have looked at how the rapid change to remote consulting precipitated by the pandemic was depicted in the media. A previous study by the present authors’ team found negative depictions of general practice and GPs in national newspapers.^6^

In the present study, the authors’ sought to examine UK newspaper coverage of remote consulting early in the pandemic and also as the first wave waned.

Research questions were:

- How did mainstream UK newspapers cover the transition from the conventional GP service to a remote (telephone or video) one?
- How was the message about remote services framed?
- What was the assumed audience?
- What metaphors and other tropes and techniques were used to convey what was happening?

Because of concerns about potential digital exclusion of certain demographic groups, particular interest was given to comparing coverage by broadsheets (aimed at highly-educated and high-income audiences), tabloids (generally aimed at less well-educated and lower-income groups), and publications marketed to Black and minority ethnic groups.

**Table:** How this fits in

Remote consulting changed UK general practice overnight, resulting in new barriers to access and levels of care. This study explored how this change was portrayed in national newspapers over time. Early newspaper coverage of this change was largely positive and emphasised its necessity for safety reasons during the pandemic. Later coverage was more negative, raising concerns about quality and safety of care and digital inequalities.

## METHOD

### Management and governance

This study was conducted between July and October 2020. It was overseen by an independent advisory group with a lay chair and a separate patient advisory group.

### Study design

A thematic content analysis of a systematic sample of newspaper articles in defined time periods, selected to provide maximum variety of media perspectives, was undertaken

### Search strategy and sampling frame

A method used successfully in a previous study of how general practice is depicted in the media was followed.^6^ The eight most widely-circulated mass-circulation national newspapers were included (Table 1), as well as *The Voice*, self-described as *‘Britain’s favourite Black newspaper’*.

**Table 1.** Number of published articles by newspaper and level of substance

**Publication**	**Period 1 (2 March–31 May 2020), *n***			**Period 2 (30 July–12 August 2020), *n***		
	**Primary dataset^a^**	**Secondary dataset^b^**	**Total**	**Primary dataset^a^**	**Secondary dataset^b^**	**Total**
*Guardian*	3	19	22	2	0	2
*Times* /*Sunday Times*	3	24	27	3	1	4
*Telegraph* /*Sunday Telegraph*	4	22	26	3	0	3
*Independent*	2	12	14	4	0	4
*Daily Mail*/*Mail on Sunday*	4	21	25	3	0	3
*Sun*	0	6	6	0	1	1
*Mirror*	0	4	4	1	0	1
*Express*	2	10	12	1	0	1
*Voice*	1	0	1	0	0	0
Total	19	118	137	17	2	19

a Articles where remote consulting was the main or a substantial focus.

b Articles where remote consulting was mentioned but not a major focus.

The lead author searched LexisNexis Academic UK (https://www.lexisnexis.com/uk/legal/news) and The Voice’s website (https://www.voice-online.co.uk) for newspaper articles during two time periods. Period 1 (2 March–31 May 2020) was chosen to include articles published during the first wave of the pandemic as cases were rising and general practice was introducing and adapting to remote services. The key search terms used were ‘GP(s)’ combined with each of seven further terms: ‘video’, ‘phone’, ‘telephone’, ‘remote’, ‘digital’, ‘online’ and ‘virtual’. All articles containing reference to remote GP consultations, in whichever form, were extracted.

This search was later repeated for period 2 (30 July–12 August 2020), chosen because it followed the announcement from Matt Hancock that remote-by-default would be long-term policy. The shorter time period was selected because, after 4 August, no articles were identified.

### Data management and analysis

After close reading, the lead author divided articles into three categories:

- articles in which remote GP consultations were the main focus;
- articles containing significant discussion about remote GP consultations, but in which this was not the main focus; and
- articles containing minor reference (usually in passing) to remote GP consultations.

The first two categories were used as the primary dataset. All authors read these articles. The number of articles in each category was tabulated by publication (daily and Sunday editions were combined).

Articles were analysed thematically using Green and Thorogood’s method.^7^ This offers a systematic approach to synthesising and coding qualitative data, and facilitates both thematic overviews and interpretation. The lead author read through each article in the first two categories twice, and each article in the third category once, making notes on recurring themes, topics, and language. Details were tabulated in Microsoft Excel along with relevant contextual information.

Codes were identified based on recurring themes, topics, and narrative devices in these articles, and sections of data, together with their article details, were grouped accordingly. As the analysis progressed, various codes were brought together into one overarching theme. The number of articles pertaining to each code, or group of codes, was then tabulated. This approach was used for both time periods, extending the framework in period 2 to incorporate some new codes.

Throughout the study, the lead author consulted the other authors, sharing examples of raw data, emerging themes, and analysis. One author reviewed the coding to ensure consistency and reviewed original sources to help guide the thematic analysis.

### Researcher perspective

This interdisciplinary study harnessed the authors different academic and professional backgrounds: one author is a humanities scholar with a doctorate in the study of intertextual influences in fiction (how novels influence the writing in other novels) and an interest in media narratives; her work on this study was one of several interdisciplinary internships established within the University of Oxford to cross-fertilise approaches from the social sciences and humanities into healthcare research. Another author is a social scientist with a doctorate in technological change; another is a sociologist of healthcare; and another is an academic GP. Key to the development of the analysis was discussion among the research team, in which different philosophical assumptions and interpretations of data were shared and negotiated. Disagreements were minor and consensus was reached by discussion.

## RESULTS

### Description of dataset

For period 1, a primary dataset of 19 articles, plus 118 additional articles that made minor reference to remote GP consultations, were identified. For period 2, the primary dataset was 17 articles, with two further articles making minor reference to the topic. The distribution of articles in the different sources is shown in Table 1. All except one national newspaper covered the topic as a main or significant focus of at least one article across both periods. Four articles in period 1 were written by GPs (in one case, the GP was also affiliated with a healthcare communications firm); in period 2 there were no articles written by GPs, but GPs and patients were widely quoted.

Themes identified in the primary dataset were widely reflected in articles in the secondary dataset. As they tended to be consistent across broadsheet and tabloid newspapers, a comparative analysis by source was not undertaken.

### Temporal context

Period 1 corresponded to the lead-up to the UK’s lockdown (in which leisure and hospitality services were closed), the lockdown period itself (23 March–23 June, where people were largely required to stay at home^8^), and the early stages of the lifting of lockdown. A phased reopening of public spaces, such as schools and shops, occurred in late June 2020,^9^ followed by hospitality, leisure, and entertainment services in July.^10^ The announcement by Hancock on 30 July 2020 that all GP consultations would be remote-by-default, unless there was a compelling clinical reason to see a clinician in person,^5^ corresponded almost exactly with the time when the public were allowed to leave their homes once more for non-essential purposes.

Thus, the initial press reaction to remote GP consultations (period 1) occurred at a time when policy on this service model was in step with wider measures to encourage physical distancing and remote working. However, the later reaction (period 2) occurred at a time when policy had abruptly become out of step with wider infection control measures.

### Findings from thematic analysis

In the primary datasets, five recurring themes, topics, and narrative devices were identified: reasons for the change; depictions of technology; war and revolution metaphors; the need for rapid change in the NHS; and trade-off between positive and negative impacts. These are summarised in Tables 2 and 3. Where a newspaper published more than one article on one day, articles have been distinguished by ‘(a)’ and ‘(b)’.

**Table 2.** Coding table for period 1 (2 March–31 May 2020)

	**G 6 Mar**	**DM 10 Mar**	**Ti 10 Mar**	**E 11 Mar**	**G 11 Mar**	**E 14 Mar**	**Te 15 Mar**	**I 16 Mar**	**Ti 17 Mar**	**DM 31 Mar**	**Ti 4 Apr**	**Te 6 Apr**	**V 9 Apr**	**DM 14 Apr**	**I 15 Apr**	**G 19 Apr**	**DM 2 May**	**Te 22 May**	**Te 25 May**	**Total**
**Reasons for change**	X	X	X	X	—	X	X	X	X	X	—	—	X	—	—	—	—	—	X	11

**Technology**																				
Bespoke innovations	X	X	—	X	X	—	—	X	—	X	—	X	X	—	X	—	—	—	X	10
Generic (Zoom, Skype)	—	—	—	X	X	X	—	—	—	—	—	—	X	X	—	—	—	X	X	7
Generic (WhatsApp)	—	—	—	—	—	—	—	—	—	—	—	—	—	—	—	—	—	—	—	0

**Metaphors**																				
War, crusade	X	X	—	X	—	—	—	X	—	—	—	—	—	X	X	—	—	—	—	6
Revolution	—	—	—	—	—	—	—	—	—	—	—	—	—	—	X	X	—	—	X	3

**Change**																				
Has been rapid	—	—	X	—	X	—	—	X	—	—	X	—	—	—	X	X	X	—	X	8
Need to modernise the NHS	—	—	—	—	—	—	—	—	—	—	—	—	—	—	—	—	—	—	—	0

**Tensions**																				
Benefits of remote	X	X	X	X	X	X	—	X	X	X	X	X	X	X	X	X	X	—	X	17
Positive patient experience	—	—	—	—	—	—	—	—	—	—	—	—	—	—	—	—	—	X	X	2
Concerns about remote	X	—	—	X	—	X	X	—	X	X	X	—	—	X	—	X	X	—	X	11
Negative patient experience	—	—	—	—	—	—	—	—	—	—	—	—	—	—	—	—	—	—	X	1

*DM =* Daily Mail. *E =* Express. *G =* Guardian. *I =* Independent. *Te =* Telegraph. *Ti =* Times. *V =* Voice.

**Table 3.** Coding table for period 2 (30 July–12 August 2020)

	**G (a)^a^ 30 Jul**	**I 30 Jul**	**G (b)^a^ 30 Jul**	**DM 30 Jul**	**Te 30 Jul**	**Ti 30 Jul**	**Ti (a)^a^ 31 Jul**	**I 31 Jul**	**Te (a)^a^ 31 Jul**	**DM 31 Jul**	**Ti (b)^a^ 31 Jul**	**E 31 Jul**	**Te (b)^a^ 31 Jul**	**I 1 Aug**	**DMir 1 Aug**	**DM 2 Aug**	**I 4 Aug**	**Total**
**Reasons for change**	—	—	X	X	—	—	—	X	—	—	—	X	—	—	—	X	—	5

**Technology**																		
Bespoke innovations	—	—	—	—	—	—	—	X	—	—	—	—	—	—	—	—	—	1
Generic (Zoom, Skype)	—	—	—	X	—	—	X	—	X	—	X	X	X	X	X	—	—	8
Generic (WhatsApp)	—	X	—	X	X	X	—	—	—	—	—	X	—	—	—	—	—	5

**Metaphors**																		
War, crusade	—	—	—	—	—	—	—	—	—	—	—	X	—	—	—	—	—	1
Revolution	—	—	—	—	—	—	—	—	—	—	—	—	X	—	—	—	—	1

**Change**																		
Has been rapid	—	—	—	—	—	—	—	—	—	—	—	—	—	—	—	—	—	0
Need to modernise the NHS	—	—	—	—	—	—	X	—	X	X	X	—	X	—	—	—	—	5

**Tensions**																		
Benefits of remote	X	X	X	X	—	X	X	X	—	X	—	—	X	X	—	—	—	10
Positive patient experience	—	—	X	—	—	—	—	—	—	—	—	—	—	—	X	—	—	2
Concerns about remote	X	X	X	X	—	—	X	X	X	X	—	X	X	X	—	—	—	11
Negative patient experience	—	—	—	—	—	—	—	—	—	—	—	—	—	X	—	X	X	3

a *(a) and (b) used to distinguish between articles published in the same publication on the same date. DM =* Daily Mail. *DMir =* Daily Mirror. *E =* Express. *G =* Guardian. *I =* Independent. *Te =* Telegraph. *Ti =* Times.

#### Reasons for the change

Most articles published at the beginning of March 2020 sought to explain why remote consultations were being introduced at a time of rapid change and uncertainty; most related to slowing the spread of the virus. Some focused on patients, depicting remote consultations as reducing *‘the risk of someone already infected with the virus spreading it further’* (*Express*, 11 March). Some articles explicitly advised, and even commanded, patients not to make in-person visits to their GP, as in *‘Ring GPs, don’t visit’* (*Telegraph*, 15 March).

Other explanations were GP-focused. Remote consultations were described not only as *‘a way of* [GPs] *protecting themselves’* (*Guardian*, 6 March) from the virus but also as a means of increasing efficiency of care, to *‘free GPs to deal with the extra workload created by the virus’* (*Guardian*, 6 March). However, almost nothing was said in the lay press about the workload associated with the task of effectively and rapidly embedding the technology in workflows or familiarising staff with the new system. A single article in the dataset reported one GP who felt that rather than increasing efficiency, his workload had increased so much that *‘he had 40 other patients to phone back’* (*Daily Mail*, 24 March).

The narrative of improved efficiency in remote primary care was occasionally reinforced in the period 2 dataset by reports of a Royal College of General Practitioners survey, which was depicted as having found that *‘seven in ten* [GPs] *said telephone appointments increased their efficiency’* (*Daily Mail*, July 30). It is noteworthy that this finding was taken from a report that predominantly questioned the remote-first policy,^11^ and that the *Daily Mail* chose not to convey the overall sense of the report.

The period 2 dataset included some retrospective explanations, with reasons appearing in the past tense. One article, which offered a negative overall assessment of the new remote-first policy going forward, nevertheless depicted it as having been justified when first introduced:

‘At the height of the COVID-19 epidemic it was understandable that GPs should try to avoid face-to-face contact where possible. It was vital the disease was contained and that doctors themselves had the best possible protection.’

(*Express*, 31 July)

The strong message communicated to the public was that the policy of defaulting to remote was justified then (since the threat of the pandemic was serious and pressing) but is no longer justified now (since the pandemic has subsided). Instead, later articles argued, there is no good reason to maintain remote as default. Rather, a return to face-to-face in some circumstances could be justified on the grounds of choice: clinicians and patients should *‘decide what works best for them’* (*Express*, 31 July).

#### Depictions of technology

Remote consulting was frequently depicted in the period 1 dataset as delivered via novel and bespoke technology, with an emphasis on innovation and private-sector entrepreneurship. Several commercial companies that had been offering remote consultations (in partnership with the NHS or privately) during the pandemic were showcased. In most cases, the narratives depicted technologies in active terms as the agents of change.

The GP Chief Medical Officer of LIVI, for example, announced that the company’s remote technology *‘allows GPs* […] *to care for people at home via digital consultations’* (*Independent*, 15 April). The language used conveys the idea of master and subordinate: the latter is incapable of acting without the permission and support of the former. The article includes firm and confident predictions with technology central to the achievement of improved outcomes, for example, *‘Digital healthcare will keep people at home and therefore save lives’*. In contrast, GPs themselves were not depicted by any articles in the period 1 dataset as active agents or as saving any lives. A symbiotic relationship between GPs and digital health care was depicted in only one article (*Voice*, 9 April), with two surgeries in Newham *‘offering remote GP consultations in collaboration with Docly’*, a text-based service described as working *‘in unison’* with practices. Rather than saving lives, its role is to *‘facilitate’* health care and *‘ease the burden on primary care’*.

Although GPs who were not linked to digital healthcare companies were widely in favour of remote consultations, there was scepticism towards the technology firms. One GP, for example, described the firms as *‘innovative (or predatory, depending on your take on it)’* (*Guardian*, 11 March).

The narrative of bespoke, heroic technologies developed by an entrepreneurial private sector did not persist. In the period 2 dataset, no representatives from private health technology companies wrote articles or provided quotes, and only one such company (Doctorlink) was mentioned briefly (*Independent*, 31 July). Instead, the new government policy was often reported as best delivered by doctors using familiar, freely available, and non-bespoke technology (such as Zoom, Skype, and/or WhatsApp). Matt Hancock is reported as encouraging healthcare professionals *‘to speak with both colleagues and patients’* (*Independent*, 30 July) via WhatsApp. In period 2, data shows that agency is restored to GPs, with generic technologies facilitating, rather than delivering, patient care.

#### War and revolution metaphors

Military metaphors were evident in the period 1 dataset. One article, for example, entitled *‘GPs told to switch to digital consultations to combat COVID-19’* (*Guardian*, 6 March), paints the image of GPs as soldiers on the frontline and coronavirus as the enemy. The military metaphor is further developed into an enemy that is gaining ground, with GPs *‘fight* [ing] *the spread of coronavirus’* (*Daily Mail*, 10 March), while public health chiefs *‘battle to reduce the risk’* of further spread (*Express*, 11 March). In an article entitled *‘Digital front opens in war on disease* […] *‘* (*Times*, 29 March), commercial start-ups are depicted as allied with doctors in a strategically-organised battle against COVID-19.

Another article talks not — as might be expected — of GPs (agents) deploying technologies (tools) but of the reverse: digital appointments helping *‘to deploy the workforce* [GPs] *more efficiently’* (*Independent*, 16 March), with technology explicitly superseding doctors in the military hierarchy.

The metaphor of revolution was also prominent in the period 1 dataset. The original, emergency move to remote consultations is described as having *‘revolutionised GP surgeries pretty much overnight’* (*Telegraph*, 25 May). However, while one article agrees that it was *‘a very rapid and necessary revolution’* (*Guardian*, 19 April), another suggests that the revolution is yet to happen (*Independent*, 15 April). This lack of consensus on whether the revolution is in the past or the future may reflect uncertainty about whether it is a technological revolution (achieved simply by installing new technology) or a service revolution (not achieved until the technology is actually in regular and unproblematic use).

The period 2 dataset paints a different picture. Despite Matt Hancock’s comparisons of the pandemic with a war in his speech,^5^ no military metaphors are mentioned in the articles, suggesting that the media considered the ‘war’ against COVID-19 to be over. Although the metaphor ‘crusade’ appears on one occasion, it is not against the virus, but is rather *‘* [Matt Hancock’s] *crusade to introduce more digital technology to the NHS’* (*Express*, 31 July). Similarly, revolution was mentioned only once in the period 2 dataset, but not linked to the pandemic or the immediate response to it. By describing Matt Hancock’s suggestion that the NHS moves towards *‘Zoom medicine’* as *‘a bold, potentially revolutionary step’* (*Telegraph* (b), 31 July), the article places the revolution firmly in the future and shifts the agency from industry and clinicians to politics and policy.

#### The need for rapid change in the NHS

Articles in the period 1 dataset depict the speed at which remote consultations were implemented as *‘astonishing’* (*Times*, 4 April), *‘dizzying’* (*Guardian*, 11 March), and *‘dramatic’* (*Guardian*, 19 April). This sense of surprise partly reflects a widespread perception of the NHS, and general practice in particular, as inherently slow and reluctant to change. Leading change agents, such as Sir John Oldham, were quoted urging their more reluctant colleagues to seize the day — *‘now is the moment for my GP colleagues to embrace video consultations’* (*Times*, 10 March).

The Chair of Council of the Royal College of General Practitioners was widely quoted on this theme, for example, *‘it is coronavirus that has propelled primary healthcare into the digital age after years of dragging its feet’* (*Telegraph*, 25 May). The coverage suggests, on the one hand, that there was no real reason for the delay apart from inertia — but on the other, that in times of crisis primary care services can adapt at a remarkable pace.

Articles in the period 2 dataset develop this narrative by expanding on Matt Hancock’s comment^5^ that the NHS must not fall back into what are depicted as bad habits (such as, inefficient routines and practices that fail to maximise the efficiency gains of technology). One article describes the NHS generally as *‘resistant to change, over-bureaucratic and its administration is often technologically backwards’* (*Telegraph* (b), 31 July). Another article comments on the *‘archaic administration of* […] *doctors’ surgeries, which can often feel like places where the modern internet fears to tread’* and depicts general practice as in need of a *‘digital reckoning’* (*Times* (b), 31 July).

#### Trade-off between positive and negative impacts

Articles in both datasets talked of benefits and harms of remote consulting for both clinicians and patients. In period 1, GPs were reported as viewing the move as *‘sensible’* (*Guardian*, 6 March; *Express*, 14 March), and mentions positive consequences including improved safety, the ability for self-isolating GPs to work from home, and convenience. As one quoted GP put it, *‘digital healthcare, if done well, has a way of creating positive change’* (*Voice*, 9 April). While GPs were also reported as concerned about hypothetical missed symptoms and diagnoses, *‘problems that require relationships’* (*Daily Mail*, 2 May), and barriers to access, especially that the elderly *‘will struggle with replacement telephone consultations’* (*Telegraph*, 15 March), the trade-off was depicted as worthwhile, with benefits discussed more frequently and emphatically.

The trade-offs of remote consulting were viewed differently in the period 2 dataset. While some benefits, such as convenience and suitability for *‘simple conditions’* (*Guardian* (b), 30 July), were acknowledged, limitations were more numerous and discussed more often. The risk of missed diagnoses, threats to the therapeutic relationship, and concerns about digital exclusion were covered in more detail with additional examples (for example, articles discussing digital exclusion now included not just the elderly but also those with learning difficulties and low-income groups). Articles in the period 2 dataset explored a growing list of clinical situations where remote consulting would be impossible or inappropriate, such as blood tests, vaccinations, and physical examinations, and sensitive situations, such as gynaecological examinations, detection of abuse, and certain mental health conditions.

The clear shift over time from an overall positive trade-off to a contingent and sometimes negative one was still apparent but less marked in patients’ accounts. Both periods included both positive and negative accounts, though the latter were more common in period 2. In the period 1 dataset, patients without serious complaints described their experiences as *‘very simple and easy’* (*Telegraph*, 25 May, video consultation) and *‘the most painless doctor’s appointment of my life’* (*Telegraph*, 22 May, phone consultation). Those quoted in period 2 with minor complaints also described *‘a positive experience’* (*Guardian* (b), 30 July, phone consultation) and considered the encounter a *‘pleasure’* (*Daily Mirror*, 1 August, phone consultation).

But even in the early days of remote consultations, negative patient experiences, especially in those with more serious complaints, were reported. One patient in the period 1 dataset is quoted as saying his GP *‘missed my coronavirus symptoms’*, leading (it was claimed) to his rapid deterioration and hospital admission (*Telegraph*, 25 May, phone consultation). Other stories in the period 2 dataset described an alleged missed cancer (*Independent*, 4 August, video consultation) and missed abdominal emergency (*Daily Mail*, 2 August, phone consultation); in the second example the patient’s wife is quoted saying *‘people will die if this lack of face-to-face consultations continues’*.

## DISCUSSION

### Summary

To the authors’ knowledge, this is the first study of newspaper coverage of the shift to remote consulting in UK general practice to identify an important change in press coverage over time. In the early weeks of the pandemic, articles depicted remote consulting positively, equating digital change with progress and novel technological solutions as driving improved efficiency and safety in a service that was overdue for modernisation. However, as the first wave of the pandemic waned, the lay press began to question the need for a remote-first policy. Problems such as missed diagnoses, difficulty assessing patients with serious or complex conditions, challenges to the therapeutic relationship, and digital inequalities, which had been mentioned as hypothetical concerns in the first period, were now substantiated with vivid first-person accounts of (alleged) actual experiences and events. The remote-first model that had been introduced with such impressive speed to respond to the pandemic was now, it seemed, interfering with clinical quality, introducing risk and curtailing patient choice.

### Strengths and limitations

An extensive database was searched using a wide range of terms, in various combinations, identifying 156 articles. The authors focused primarily on the 36 articles with greatest narrative richness and triangulated these against the wider dataset. The study of two defined time periods enabled the authors to uncover and chronicle shifting depictions of the service over time.

Newspaper articles may not accurately represent all the information that was provided to the public regarding the shift, or accurately reflect the public’s perception of remote GP consultations. Furthermore, despite multiple searches on LexisNexis Academic UK database, there is a risk that some articles might have been missed. Another limitation is that this study was (for resource reasons) restricted to the UK.

### Comparison with existing literature

To the authors’ knowledge, no other published studies have investigated media portrayals of the shift to remote consultations. Policy announcements in the early weeks of the pandemic placed heavy reliance on technological measures to maximise infection control, though unlike lay media articles, these acknowledged that the change would be difficult and challenging.^1^^,^^12^ Editorials in the academic literature early in the pandemic flagged the crisis in hopeful terms as an opportunity to achieve overdue modernisation of services and efficiency improvements.^13^^–^^17^ Empirical evaluations of new telehealth services were quickly produced, and largely emphasised the benefits of such services.^18^^–^^21^ Review articles and commentaries depicted such services as part of a wider digital response to the pandemic, which also included new and repurposed technologies for population surveillance, case identification, contact tracing, point-of-care diagnosis, and disease monitoring.^22^^,^^23^ In short, policymakers and researchers as well as journalists appear to have placed considerable faith in the power of technology to respond to the virus.

During the same period, the clinical literature published practical guides to assist those implementing remote services.^24^ Clinical editorials and commentaries documented GPs’ concerns that some core functions of primary care, especially relating to the therapeutic relationship and containment of clinical risk, risked being compromised.^25^^–^^29^

Qualitative research of the experience of remote consultations suggest that the concerns raised by the lay press in the period 2 dataset are, overall, well founded. These studies have documented greater convenience (for some), technological challenges, and higher demands on the patient than in face-to-face encounters.^30^^–^^32^ Others have written on the potential for exacerbating health inequalities through various kinds of digital exclusion.^33^ An earlier study by the present authors’ team found that patients felt strongly that they should be able to choose a remote consultation.^32^

### Implications for research and practice

The present study was limited to media coverage in the UK in the context of a rapid policy initiative to remote consulting in that country. Further studies of media coverage in other countries, which also pushed remote GP consultations as a pandemic response,^34^ might provide contrasts and additional insights. A comparative study of media coverage aimed at older people in the UK, Australia, and US in the 2 weeks following the announcement of the pandemic (11 March 2020) showed that while media in three countries depicted telehealth positively, there were significant differences that the authors attributed to differences in geography, the nature and funding of health services, and how older people and their use of technologies are understood in society.^35^

As the first wave of the pandemic came and went, media depictions of remote consulting appear to have evolved from an ‘efficiency and safety’ narrative to a ‘risks, inequalities, and lack of choice’ narrative. The authors’ suggest that, especially given the negative portrayals of general practice by the media documented previously,^6^ there is an urgent need to restore public trust in general practice in general, and remote consulting in particular. Three measures could help.

First, clarification is needed on what kind of clinical consultations with what kind of patient are suited to what kind of medium (a topic that the authors are currently researching). Second, rather than any medium being imposed ‘by default’, the wide menu of consulting options now available in general practice should be provided flexibly and with sensitivity to patients’ needs and preferences. The kind of consultation offered should be decided on the basis of what is best for the patient; where possible, this should be a shared decision. Finally, measures must be taken to assure safety (for example, through better guidance, standards, and training) and avoid inequity (for example, by making face-to-face appointments clearly available and accessible to all for whom remote consultations are unacceptable or inappropriate).
